# Cost-effective treatment of ocular surface squamous neoplasia for an undocumented and uninsured New York City patient: a case report

**DOI:** 10.1186/s13256-020-02510-w

**Published:** 2020-10-02

**Authors:** Norman A. Saffra, Trisha S. Emborgo, Codrin E. Iacob, David S. Kirsch

**Affiliations:** 1 Department of Ophthalmology, St. John’s Episcopal Hospital, Far Rockaway, NY USA; 2 Department of Ophthalmology, New York Eye and Ear Infirmary of Mount Sinai, New York, NY USA; 3 Department of Pathology, New York Eye and Ear Infirmary of Mount Sinai, New York, NY USA

**Keywords:** Ocular surface squamous neoplasia, Undocumented, Uninsured, Cost-effective treatment, Case report

## Abstract

**Background:**

New York City has a heterogeneous population with many undocumented and uninsured immigrants from equatorial areas who have a higher incidence of ocular surface squamous neoplasia. To the best of our knowledge, this is the first documented selection of this cost-effective treatment of ocular surface squamous neoplasia (the use of absolute ethanol along the corneal margin, primary excision, double freeze-thaw cryopexy, and primary conjunctival closure) for an undocumented and uninsured New York City patient.

**Case presentation:**

A 35-year-old man from Ecuador presented to a New York City emergency department due to worsening discomfort of a long-standing left eye pterygium. A slit-lamp examination of the left eye demonstrated a nasally located conjunctival mass measuring 6 × 8 mm extending onto the cornea (3 mm superiorly and 6 mm inferiorly on the cornea). Histological diagnosis confirmed squamous cell carcinoma *in situ* arising from the pterygium. Surgical excision with adjunctive absolute alcohol with additive double freeze-thaw cryopexy was performed. Our patient has remained free of tumor recurrence at year 2 postoperative visit.

**Conclusions:**

Our case highlights the need to choose a cost-effective treatment for ocular surface squamous neoplasia in an at-risk population among undocumented and uninsured patients. Areas in the world with similar types of populations or treatment challenges may need to consider this approach as a primary treatment option.

## Background

Ocular surface squamous neoplasia (OSSN) includes a variety of dysplastic changes of the conjunctiva and cornea, ranging from benign dysplasia to carcinoma *in situ* to invasive squamous cell carcinoma [1, 2]. Risk factors include ultraviolet (UV) light, immunosuppression, human immunodeficiency virus (HIV), human papillomavirus (HPV), mutations of p53, and older age [1–3]. Patients typically complain of redness, foreign body sensation, and growth on the ocular surface [1].

Treatment of OSSN includes a variety of options and even combinations of therapy, such as excisional biopsy, cryotherapy, or topical chemotherapy [4, 5]. Personalizing treatment requires evaluation of not just the medical aspects of the condition but also the social needs of the patient. For example, the use of primary or adjunctive topical chemotherapy may not be ideal for an undocumented and uninsured New York City (NYC) patient. The potentially high out-of-pocket costs for topical chemotherapy, lost work time for follow-up visits, the cost of the office visits, and even transportation costs to the visits, may limit a patient’s ability to comply with topical chemotherapy regimens. With these factors in mind, we report the first documented selection of this cost-effective treatment of OSSN (the use of absolute ethanol along the corneal margin, primary excision, double freeze-thaw cryopexy, and primary conjunctival closure) for an undocumented and uninsured NYC patient.

## Case presentation

A 35-year-old man from Ecuador presented to a NYC emergency department due to worsening discomfort of a long-standing left eye pterygium. He denied changes in vision and discharge to both eyes. Further history revealed he is an undocumented and uninsured outdoor day-laborer from Ecuador and former tobacco smoker (he quit 8 years ago, two to three cigarettes per day). He denied significant past medical history, surgical history, and family history of malignancy. Physical examinations were unremarkable apart from the eye lesion. Documented serological HIV testing was performed and confirmed that he is HIV-negative.

**Fig. 1:**
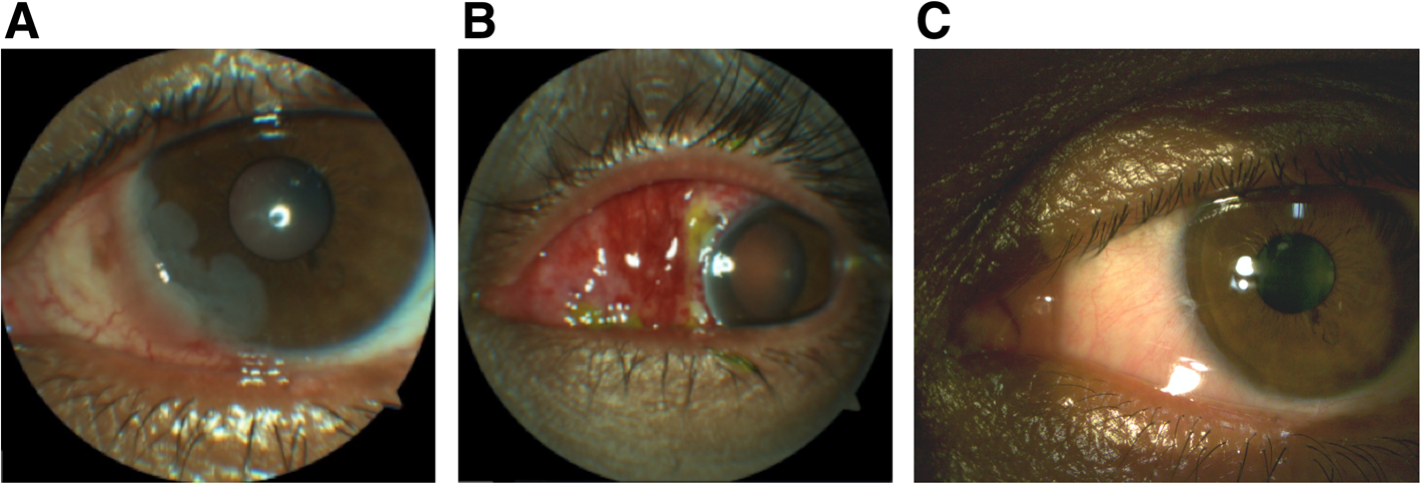
Clinical appearance of the left eye. **a** At presentation, a triangular-shaped tissue that pulled toward the cornea showed a 6 × 8 mm elevated flesh-like mass, 2+ injection, and lobulated gelatinous extensions of the conjunctival mass encroaching the cornea (3 mm superiorly and 6 mm inferiorly on the cornea). Absolute ethanol was used intraoperatively along the nasal corneal margin prior to incision. **b** Postoperative day 1 showed expected conjunctival chemosis and surgical clearance of the gelatinous material over the cornea. **c** Postoperative year 2 showed 1+ conjunctival injection, trace conjunctival scarring, and no tumor recurrence

His visual acuity without correction was 20/20 in the right eye and 20/25 in the left eye. His pupils were 5–2 mm with no apparent pupillary defect in both eyes. Extraocular muscles were intact in both eyes. A slit-lamp examination of the right eye was unremarkable. A slit-lamp examination of the left eye (Fig. 1a) demonstrated a 6 × 8 mm elevated flesh-like mass, 2+ injection, and lobulated extensions of the conjunctival mass encroaching the cornea (3 mm superiorly and 6 mm inferiorly on the cornea). Tonometry applanation was within normal limits: 16 mmHg right eye and 15 mmHg left eye at 07:20 a.m. Dilated funduscopic examination of both eyes was unremarkable.

Surgical excision with adjunctive absolute alcohol with additive double freeze-thaw cryopexy was performed. Using a “no-touch” technique, the clinical boundaries of the tumor were outlined by adding approximately 4 mm margin of clinically normal tissue on the superior, inferior, and temporal margins [6]. The conjunctiva was marked with a cautery, then absolute alcohol-soaked Weck-Cels® were placed for 10 seconds each along the temporal, superior, inferior, and nasal margins of the tumor which extended into clear cornea (Shields C.L., The number of seconds for the application of the alcohol-soaked Weck-Cel, personal email correspondence on 26 July 2020) [6]. Once the epithelium was loose and using a Weck-Cel, the epithelial portion of the tumor was removed without violating Bowman’s membrane [6]. Conjunctiva extension was then excised starting from the caruncle and undermining it toward the limbus with the removal of the tumor in a single block. The specimen was marked and sent for pathology. Cautery was applied to the base, which was followed by Weck-Cel soaked with absolute alcohol applied to the base [6]. A No. 57 Beaver® blade was then used to scrape away all limbal cells and all remaining limbal tissue [6]. An additional application of a Weck-Cel soaked in absolute alcohol was applied [6]. The conjunctiva was undermined and then closed with interrupted 6 × 7-0 Vicryl sutures after double freeze-thaw conjunctival cryopexy had been applied along the entire perimeter [6]. A collagen shield soaked in an antibiotic was placed on the left eye and cycloplegic drops were placed in the left eye. A patch and shield were placed over the left eye. No topical chemotherapy was used.

**Fig. 2:**
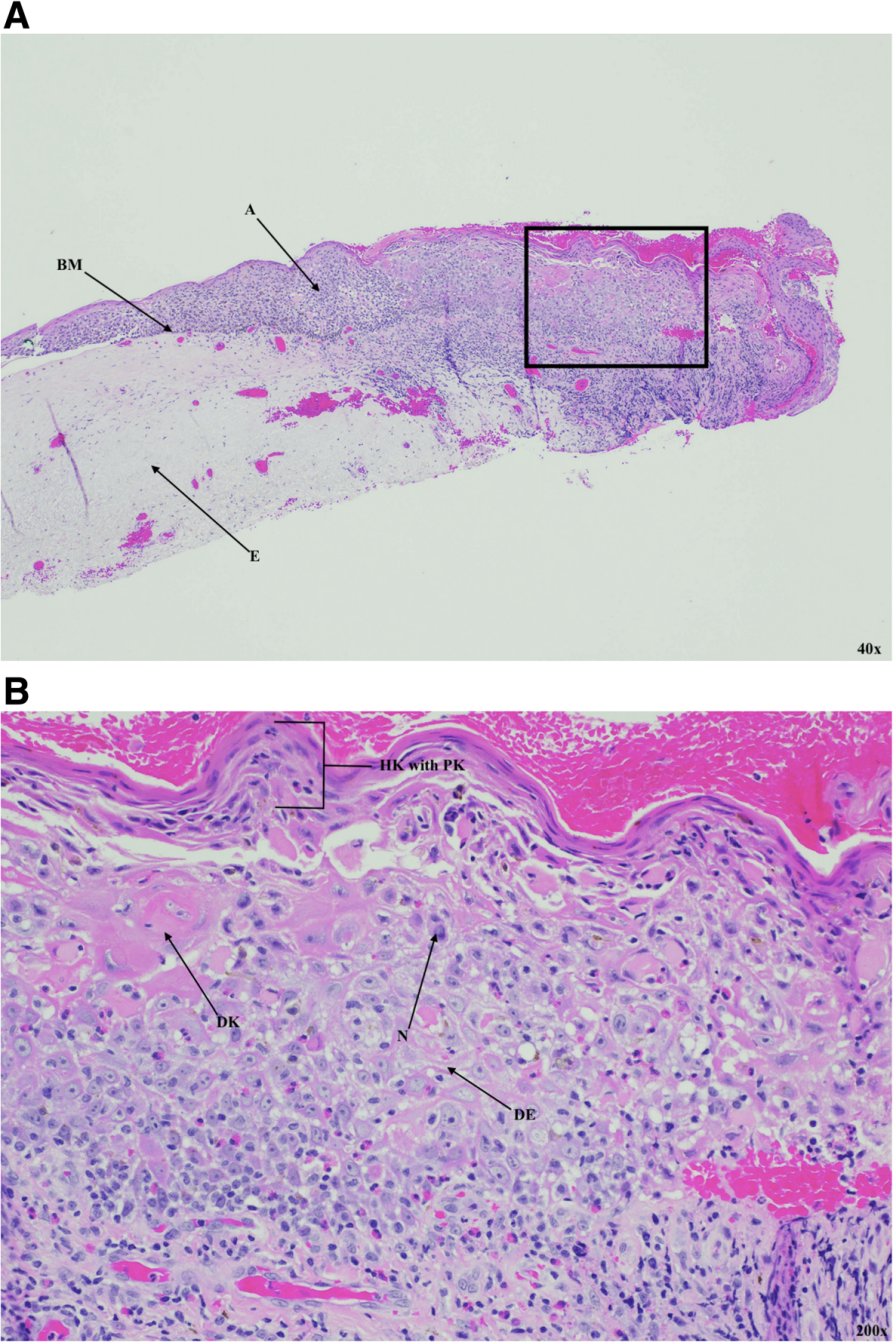
Squamous cell carcinoma *in situ*. **a**, **b** Left eye conjunctival specimen demonstrated significant acanthosis (*A*) with full-thickness dysplasia and surface keratin accumulation using hematoxylin and eosin-stained paraffin (original magnification, **a** × 40; **b** × 200). Underlying subepithelium showed elastotic degeneration (*E*) and moderate accompanying chronic inflammation. *A* acanthosis, *BM* basement membrane, *DE* desmosomes, *DK* dyskeratotic cells, *E* elastotic degeneration, *HK with PK* hyperkeratotic with parakeratotic cells, *N* enlarged nuclei

Histological diagnosis confirmed squamous cell carcinoma *in situ* arising from pterygium (Fig. 2A, B). The underlying subepithelium showed elastotic degeneration (Fig. 2A) and moderate accompanying chronic inflammation. Nasal, superior, and inferior conjunctival margins were negative for malignancy, while the corneal margin was involved by carcinoma. Immunohistochemistry was performed to test for HPV p16 and *in situ* hybridization was performed to test for HPV 6/11 and HPV 16/18. The specimen was HPV-negative.

He was discharged on the same day of the procedure. He was followed up on postoperative day 1 at an out-patient office (Fig. 1b). A slit-lamp examination on postoperative day 1 showed expected conjunctival chemosis and surgical clearance of the gelatinous material over the cornea. He was provided with medication samples of an antibiotic and corticosteroid ophthalmic suspension during this visit. Follow-up visits on postoperative week 1 and postoperative month 1 demonstrated 3+ conjunctival injection, expected corneal epithelial defect, and sutures intact. The medication samples were tapered after postoperative month 1. Postoperative month 3 showed 1+ conjunctival injection, clear cornea, sutures intact, and no recurrence of tumor. Postoperative month 7 showed 1+ conjunctival injection, trace conjunctival scarring, and clear cornea. Postoperative year 2 showed trace conjunctival scarring (Fig. 1c). He has remained free of tumor recurrence at year 2 postoperative visit.

## Discussion and conclusions

OSSN includes a variety of dysplastic changes in the conjunctiva and cornea. Risk factors include UV light, immunosuppression, HIV, HPV, mutations of p53, and older age [1–3]. The gold standard for diagnosis of OSSN is histology, while Rose Bengal, lissamine green, and methylene blue stains are non-invasive methods of clinically identifying suspicious OSSN lesions [7–9]. Treatment options include excisional biopsy, cryotherapy, and topical chemotherapy [4, 5]. To the best of our knowledge, this is the first documented selection of this cost-effective treatment of OSSN (the use of absolute ethanol along the corneal margin, primary excision, double freeze-thaw cryopexy, and primary conjunctival closure) for an undocumented and uninsured NYC patient.

UV light is known to cause DNA damage and create pyrimidine dimers [1]. Newton *et al.* studied the effects of ambient UV light on the incidence of ocular squamous cell carcinoma [10]. They found that in each 10-degree increase in latitude, the incidence of ocular squamous cell carcinoma decreased by 49% [10]. For comparison, Uganda had more than 12 cases/million per year versus the UK’s < 0.2 cases/million per year [10]. Our patient was an outdoor day-laborer from Ecuador, and UV light may have been a contributing cause of his OSSN.

Previous studies have shown that patients with OSSN younger than 50 years of age should warrant HIV testing [3]. Mahomed and Chetty reported that 12 out of 17 (70.6%) South African patients with OSSN tested for HIV had positive results and the 12 patients who were HIV-positive were all under the age of 50 years [3]. Karcioglu and Wagoner noted that OSSN in younger African patients (age range, 32–37 years) was strongly associated with HIV-positive status [11]. Pradeep *et al.* found six out of 21 (28.6%) patients with OSSN to be HIV-positive in South India [12]. Among patients who were HIV-positive in South Africa, the median age of presentation was 36 years, while patients who were HIV-negative had a median age of 54 years [12]. Guech-Ongey *et al*. found 15 US patients who were HIV-positive with squamous cell carcinoma of the conjunctiva; out of the 15, 10 (67%) were under the age of 50 years relative to acquired immune deficiency syndrome (AIDS) onset [13]. In the USA, the mean age of presentation of OSSN is 64 years [14, 15]. Our case report presents an OSSN in a 35-year-old man in the USA with documented serological HIV-negative test results and demonstrates that a young US man who is HIV-negative may still have a possible malignant ocular surface lesion.

HPV and its correlation to OSSN has been discordant. HPV incidence rate in OSSN can range from 0 to 88.1% [16–22]. Certain studies have noted HPV, specifically high-risk HPV type 16, to be highly correlated with OSSN [16]. Carrilho *et al.* found 11 out of 19 (57.9%) biopsies of the conjunctiva of patients with suspected eye cancer tested positive for HPV infection, while Galor *et al*. found 21 out of 27 (78%) OSSN specimens with HPV [17, 18]. Yet other studies, such as Manderwad *et al*., Guthoff *et al*., Sen *et al.*, and Tuppurainen *et al*., found a zero incidence rate of HPV in OSSN [19–22]. Our case is negative for p16 by immunohistochemistry and does not demonstrate a reaction to HPV 6/11 or to HPV 16/18 by *in situ* hybridization.

While tobacco smoking correlates with other squamous cell carcinomas, studies have shown that tobacco smoking does not increase the risk of the development of OSSN [15, 23, 24]. Early *et al.* found no statistically significant relationship between high-grade dysplasia and cigarette smoking, similar to Gichuhi *et al*.’s association between OSSN and cigarette smoking [15, 23]. Another study considered not only tobacco smoke but also household smoke from cooking fires; neither factor increased the risk of OSSN [24]. Although our patient was a former cigarette smoker and smoked 2–3 cigarettes per day, it was unlikely that his OSSN developed due to cigarette smoking.

Mutations or deletions of p53 have been associated with OSSN specimens [3]. p53, a tumor suppressor protein, is located on chromosome 17p13 and causes cell cycle arrest at G1-S checkpoint [25]. Mutations or deletions of p53 aid genomic instability and allow the proliferation of carcinogenesis [26]. Other risk factors, such as HPV and UV light, synergistically affect OSSN proliferation [27, 28]. Mahomed and Chetty found 28 out of 40 (70%) lesions to contain positive staining for p53 in neoplastic cells [3]. Our patient’s specimen did not receive immunostaining for p53 due to the diagnostic test not being routinely performed for a clinical squamous cell carcinoma *in situ* specimen.

The intraocular spread of OSSN and metastasis may occur but are uncommon [1]. Tumor cells can enter through the limbus and invade the Schlemm’s canal, the trabecular meshwork, the anterior chamber, the suprachoroidal space, and the uvea [1]. The invasion may cause inflammation, iritis, glaucoma, retinal detachment, and scleral thinning [1]. Although OSSN metastasis rate is < 1%, physical examinations of the preauricular/submandibular/cervical lymph nodes, parotid glands, lungs, and bones need to be conducted [1, 29]. Lee and Hirst noted that the major factor for the metastatic spread was a delay in seeking medical treatment [1]. With our patient’s inconsistent follow-up with his previous ophthalmologists, it was important to perform a thorough neck examination. Our patient’s slit-lamp examination (aside from the OSSN), intraocular pressure, and dilated funduscopic examination were unremarkable. He had no clinical evidence of anterior cervical and posterior cervical lymphadenopathy. A physical examination of his neck and musculoskeletal system were unremarkable. Thoracic and abdominal physical examinations were within normal limits, and a preoperative chest X-ray demonstrated only one calcified granuloma in the upper lobe of his right lung. Since the rate of OSSN metastasis is < 1% and unlikely in this case, based on his physical examination, no additional testing was performed; however, periodic clinical follow-up was recommended to our patient.

The standard of care for OSSN is currently evolving. Numerous studies have tested topical chemotherapy, such as 5-fluorouracil (5-FU), mitomycin C (MMC), and interferon α-2b (IFNα2b), for OSSN [5, 30]. Joag *et al.* identified that 82% of cases of OSSN had a complete response to 5-FU as the primary treatment without long-term complications [31]. Side effects of 5-FU include pain, tearing, photophobia, itching, swelling, and infection [31]. Shields *et al*. found that the recurrence rate of IFNα2b monotherapy showed a complete response of 75%, while IFNα2b with surgical excision of OSSN showed complete control in 95% of cases [32]. Besley *et al*. noted that 84.9% of patients with OSSN treated with only MMC did not have a recurrence [33]. The adverse effects of MMC include pain, epitheliopathy, allergic conjunctivitis, hyperemia, punctal stenosis, and ectropion [5]. Since IFNα2b has a minimal side effect profile in comparison to 5-FU and MMC, it would have been ideal to use IFNα2b as an adjuvant to surgical excision for our patient.

Although implementing topical chemotherapy would have been ideal and preferable to decrease the recurrence rate, socioeconomic factors and cost of treatment had to be considered. NYC is considered a sanctuary city in which undocumented immigrants are protected from detention if based only on immigration status [34]. NYC has two bills that reduce the presence of Immigration and Customs Enforcement (ICE) at Rikers Island and all City facilities [35]. With NYC being a common ground for at least 560,000 undocumented immigrants, approximately 200,000 of the undocumented NYC patients do not have access to health insurance [36, 37]. As many as 9.5% of those under the age of 65 do not have health insurance in NYC [38]. Hispanics, including persons from Mexico, Central America, South America, and the Caribbean, comprise the highest number of undocumented immigrants in the USA [39]. With NYC being a sanctuary city for undocumented immigrants, the inequality in health care access can be reflected in recent events, including the increased mortality rate of the Hispanic community due to COVID-19 in NYC [40]. Socioeconomic barriers to health care include lack of access to testing, increased co-morbidities due to decreased access to physicians, language barriers, unequal access to higher education, lack of transportation to appointments, lack of access to child care, and crowded housing [41]. Due to the limited access to primary care and specialist physicians, many undocumented and uninsured patients utilize the emergency department as their primary method of care [37]. Our patient, for example, required in-patient admission through the emergency department to obtain preoperative clearance for his OSSN excision.

In addition to using the emergency department as a method of preparing our patient for his OSSN surgery, out-of-pocket out-patient treatments (not covered by his in-patient hospital stay), such as cost of topical chemotherapy and future appointments, present as a challenge to the undocumented and uninsured. Al Bayyat *et al.* noted that out-of-pocket costs for IFNα2b can range between approximately $240 and $600 per month in the USA, while 5-FU and MMC cost $38–$75 and $100–$200 per bottle, respectively [5, 7]. Other socioeconomic factors, such as work schedules, payments for office visits, and transportation costs may limit a patient’s ability to comply with topical chemotherapy regimens. Topical chemotherapy necessitates consistent follow-up from the patient due to its specific scheduled regimens. For example, IFNα2b requires the drops to be used four times a day until clinical resolution, 5-FU is used four times a day for a week followed by a 3-week break, and MMC is used four times a day for a week followed by 2–3 weeks off [5]. Although the number of follow-up appointments for the surgical approach and for the topical chemotherapy approach may be similar, topical chemotherapy was not an option for our patient due to the high out-of-pocket medication costs and previous poor appointment adherence. He had a history of poor appointment adherence with his previous ophthalmologists due to his inability to pay for office visits and transportation costs. He also worked as an undocumented outdoor day-laborer and was unable to take time off work for his out-patient appointments. His delayed presentation to the emergency department due to limited financial means (outdoor day-laborer and uninsured), inconsistent follow-up with his physicians, and high out-of-pocket medication costs did not make him an ideal topical chemotherapy candidate. Therefore, the most cost-effective and efficacious treatment of choice for our patient was the use of absolute ethanol along the corneal margin, primary excision, double freeze-thaw cryopexy, and primary conjunctival closure [6].

In conclusion, NYC has a heterogeneous population with many undocumented and uninsured immigrants from equatorial areas that have a higher incidence of OSSN. Our patient’s day-laborer status is typical of undocumented workers in NYC and other US areas that are highly populated with undocumented immigrants [42]. Our case is the first to document the use of absolute ethanol along the corneal margin, primary excision, double freeze-thaw cryopexy, and primary conjunctival closure, as the preferred cost-effective treatment of choice for an undocumented and uninsured NYC patient. Our patient has remained free of tumor recurrence at year 2 postoperative visit. While topical chemotherapy, with or without surgery, is an evolving therapeutic option, the costs and required follow-ups can be a barrier for many patients. Areas in the world with similar types of populations or treatment challenges may need to consider this approach as a primary treatment option.

## Data Availability

Not applicable.

## References

[1] Lee GA, Hirst LW (1995). Ocular surface squamous neoplasia. Surv Ophthalmol.

[2] Mittal R, Rath S, Vemuganti GK (2013). Ocular surface squamous neoplasia - Review of etio-pathogenesis and an update on clinico-pathological diagnosis. Saudi J Ophthalmol.

[3] Mahomed A, Chetty R (2002). Human immunodeficiency virus infection, Bcl-2, p53 protein, and Ki-67 analysis in ocular surface squamous neoplasia. Arch Ophthalmol.

[4] Li AS, Shih CY, Rosen L (2015). Recurrence of ocular surface squamous neoplasia treated with excisional biopsy and cryotherapy. Am J Ophthalmol.

[5] Al Bayyat G, Arreaza-Kaufman D, Venkateswaran N (2019). Update on pharmacotherapy for ocular surface squamous neoplasia. Eye Vis (Lond).

[6] Shields JA, Shields CL, De Potter P (1997). Surgical management of conjunctival tumors. The 1994 Lynn B. McMahan Lecture. Arch Ophthalmol.

[7] Sayed-Ahmed IO, Palioura S, Galor A, Karp CL (2018). Diagnosis and medical management of ocular surface squamous neoplasia. Expert Rev Ophthalmol.

[8] Kim J, Foulks GN (1999). Evaluation of the effect of lissamine green and rose bengal on human corneal epithelial cells. Cornea.

[9] Steffen J, Rice J, Lecuona K, Carrara H (2014). Identification of ocular surface squamous neoplasia by *in vivo* staining with methylene blue. Br J Ophthalmol.

[10] Newton R, Ferlay J, Reeves G, Beral V, Parkin DM (1996). Effect of ambient solar ultraviolet radiation on incidence of squamous-cell carcinoma of the eye. Lancet.

[11] Karcioglu ZA, Wagoner MD (2009). Demographics, etiology, and behavior of conjunctival squamous cell carcinoma in the 21st century. Ophthalmology.

[12] Pradeep TG, Gangasagara SB, Subbaramaiah GB (2012). Prevalence of undiagnosed HIV infection in patients with ocular surface squamous neoplasia in a tertiary center in Karnataka, South India. Cornea.

[13] Guech-Ongey M, Engels EA, Goedert JJ (2008). Elevated risk for squamous cell carcinoma of the conjunctiva among adults with AIDS in the United States. Int J Cancer.

[14] Tunc M, Char DH, Crawford B, Miller T. Intraepithelial and invasive squamous cell carcinoma of the conjunctiva: analysis of 60 cases. Br J Ophthalmol. 1999;98–103. 10.1136/bjo.83.1.98 PMC1722787 10209445

[15] Gichuhi S, Sagoo MS, Weiss HA, Burton MJ (2013). Epidemiology of ocular surface squamous neoplasia in Africa. Tropical Med Int Health.

[16] McDonnell JM, McDonnell PJ, Sun YY (1992). Human papillomavirus DNA in tissues and ocular surface swabs of patients with conjunctival epithelial neoplasia. Invest Ophthalmol Vis Sci.

[17] Carrilho C, Gouveia P, Yokohama H (2013). Human papillomaviruses in intraepithelial neoplasia and squamous cell carcinoma of the conjunctiva: A study from Mozambique. Eur J Cancer Prev.

[18] Galor A, Garg N, Nanji A (2015). Human papillomavirus infection does not predict response to interferon therapy in ocular surface squamous neoplasia. Ophthalmology.

[19] Manderwad GP, Kannabiran C, Honavar SG (2009). Lack of association of high-risk human papillomavirus in ocular surface squamous neoplasia in India. Arch Pathol Lab Med.

[20] Guthoff R, Marx A, Stroebel P (2009). No evidence for a pathogenic role of human papillomavirus infection in ocular surface squamous neoplasia in Germany. Curr Eye Res.

[21] Sen S, Sharma A, Panda A (2007). Immunohistochemical localization of human papilloma virus in conjunctival neoplasias: A retrospective study. Indian J Ophthalmol.

[22] Tuppurainen K, Raninen A, Kosunen O, Kankkunen JP, Kellokoski J, Syrjänen S (1992). Squamous cell carcinoma of the conjunctiva. Failure to demonstrate HPV DNA by *in situ* hybridization and polymerase chain reaction. Acta Ophthalmol (Copenh).

[23] Early AD, Adelson S, Miller CJ, Mauger TF (2018). Lack of relationship between cigarette smoking and alcohol use with dysplasia grade in ocular surface squamous neoplasia. Clin Ophthalmol.

[24] Waddell K, Kwehangana J, Johnston WT, Lucas S, Newton R (2010). A case-control study of ocular surface squamous neoplasia (OSSN) in Uganda. Int J Cancer.

[25] McBride OW, Merry D, Givol D (1986). The gene for human p53 cellular tumor antigen is located on chromosome 17 short arm (17p13). Proc Natl Acad Sci U S A.

[26] Meek DW (2009). Tumour suppression by p53: a role for the DNA damage response?. Nat Rev Cancer.

[27] Ateenyi-Agaba C, Dai M, Le Calvez F (2004). TP53 mutations in squamous-cell carcinomas of the conjunctiva: evidence for UV-induced mutagenesis. Mutagenesis.

[28] Gichuhi S, Ohnuma S, Sagoo MS (2014). Pathophysiology of ocular surface squamous neoplasia. Exp Eye Res.

[29] Shields CL, Chien JL, Surakiatchanukul T, Sioufi K, Lally SE, Shields JA (2017). Conjunctival tumors: Review of clinical features, risks, biomarkers, and outcomes—The 2017 J. Donald M. Gass Lecture. Asia Pac J Ophthalmol (Phila).

[30] Viani GA, de Fendi LI (2017). Adjuvant treatment or primary topical monotherapy for ocular surface squamous neoplasia: a systematic review. Arg Bras Oftalmol.

[31] Joag MG, Sise A, Murillo JC (2016). Topical 5-fluorouracil 1% as primary treatment for ocular surface squamous neoplasia. Ophthalmology.

[32] Shields CL, Kaliki S, Kim HJ (2013). Interferon for ocular surface squamous neoplasia in 81 cases: outcomes based on the American Joint Committee on Cancer classification. Cornea.

[33] Besley J, Pappalardo J, Lee GA (2014). Risk factors for ocular surface squamous neoplasia recurrence after treatment with topical mitomycin C and interferon alpha-2b. Am J Ophthalmol.

[34] Nolo. As an undocumented immigrant, am I safer from deportation in a sanctuary city? Available at: https://www.nolo.com/legal-encyclopedia/as-an-undocumented-immigrant-am-i-safer-from-deportation-in-a-sanctuary-city.html. Accessed 12 Mar 2020.

[35] Mark-Viverito M, Dromm D, Espinal R, et al. NYC Bill Summaries for Intro. 486 & 487. Federation For American Immigration Reform. 2014. Available at: https://www.fairus.org/legislation/state-local-legislation/nyc-bill-summaries-intro-486-487. Accessed 11 Mar 2020.

[36] Mayor’s Office of Immigrant Affairs (2018). State of Our Immigrant City. MOIA Annual Report.

[37] Berlinger N, Calhoon C, Gusmano MK, Vimo J. Undocumented immigrants and access to health care in New York City: The Hastings Center; 2015. p. 1–20. Available at https://www.thehastingscenter.org/wp-content/uploads/Undocumented-NYC-Hastings-NYIC-report_final.pdf. Accessed 23 July 2020.

[38] QuickFacts New York City, New York. United States Census Bureau. 2018. Available at: https://www.census.gov/quickfacts/newyorkcitynewyork. Accessed 11 Mar 2020.

[39] Passel JS, Cohn D’V. U.S. unauthorized immigrant total dips to lowest level in decade: Pew Research Center’s Hispanic Trends Project; 2018. Available at: https://www.pewresearch.org/hispanic/2018/11/27/u-s-unauthorized-immigrant-total-dips-to-lowest-level-in-a-decade/. Accessed 26 July 2020.

[40] Vazquez J. Hispanic community in NYC ‘disproportionately’ impacted by COVID-19: Officials: NBC New York; 2020. Available at: https://www.nbcnewyork.com/news/coronavirus/hispanic-community-in-nyc-disproportionately-impacted-by-covid-19-officials/2365896/. Accessed 26 July 2020.

[41] Centers for Disease Control and Prevention (2020). Health equity considerations & racial & ethnic minority groups. Coronavirus disease.

[42] Workplace Fairness. Day Laborers. 2020. Available at: https://www.workplacefairness.org/day-laborers#1. Accessed 27 July 2020.

